# Bee Pollen and Bee Bread as a Source of Bacteria Producing Antimicrobials

**DOI:** 10.3390/antibiotics10060713

**Published:** 2021-06-13

**Authors:** Karolina Pełka, Randy W. Worobo, Justyna Walkusz, Piotr Szweda

**Affiliations:** 1 Department of Pharmaceutical Technology and Biochemistry, Faculty of Chemistry, Gdańsk University of Technology, ul. G. Narutowicza 11/12, 80-233 Gdańsk, Poland; karolina.pelka@pg.edu.pl (K.P.); justyna.walkusz@pg.edu.pl (J.W.); 2 Department of Food Science, Cornell University, Ithaca, NY 14853, USA; rww8@cornell.edu

**Keywords:** bee bread, bee pollen, *Bacillus* spp., enzymes

## Abstract

The principal objective of the study was the isolation and identification of bacteria that are present in mature bee bread (BB) and dried (ready for selling and consumption) bee pollen (BP). Obtained isolates were screened for their potential to inhibit select human pathogenic bacteria and their ability to produce enzymes of particular industrial importance. Four and five samples of BP and BB, respectively, were used for the study. In total, 81 strains of bacteria were isolated, and 34 (42%) of them exhibited antagonistic interactions with at least one reference strain of pathogenic bacteria, namely *Staphylococcus aureus* ATCC 25923, *Staphylococcus aureus* ATCC 29213, *Staphylococcus epidermidis* 12228, *Pseudomonas aeruginosa* ATCC 27857, and *Escherichia coli* ATCC 25922. The sequencing of the 16S rRNA gene revealed that all strains producing antimicrobials belong to the genus *Bacillus* spp., and among them, five species were identified: *B. pumilus* (*n* = 17), *B. altitudinis* (*n* = 9), *B. licheniformis* (*n* = 4), *B. subtilis* (*n* = 2), and *B. safensis* (*n* = 1). Furthermore, 69, 54, 39, and 29 of the strains exhibited lipolytic, proteolytic, cellulolytic, and esterolytic activity, respectively. Alpha amylase and beta galactosidase activity were rarely observed, and none of the strains produced laccase. The outcomes of the study revealed that BP and BB can be considered potential sources of bacteria producing antimicrobial agents and/or enzymes of particular industrial importance. Of course, additional research is required to verify this hypothesis, but the results of preliminary studies are promising.

## 1. Introduction

Since ancient times, honey bee products, particularly honey and propolis, have been used as traditional remedies. Both these product exhibit high antimicrobial activity and have mostly been applied for treatment of infected and difficult to heal wounds. As in the case of many other natural products, the investigation of their antibacterial/antifungal potential was impeded by the discovery of antibiotics. However, renewed interest in the investigation and use of the pharmacological (not only antimicrobial) potential of bee products has been observed within the last couple of decades. Recent studies performed with modern analytical techniques and using in vitro and in vivo models have proved that the chemical components of bee products exhibit a broad range of health-beneficial properties including antimicrobial, antioxidant, anti-inflammatory, anti-cancer, and immunomodulatory activity [1,2,3,4,5]. It has been found that the enzymatic production (by glucose oxidase (GOx)) of hydrogen peroxide is a dominant mechanism by which honey collected by bees from most plant sources exert bacteriostatic and bactericidal activity. The physiochemical properties of these products, namely high osmotic pressure and low pH, as well as some of their components, e.g., polyphenols and bee defensin−1, only support the antimicrobial effect of H_2_O_2_ [1,6,7]. Thus, these honeys are called peroxide honeys. However, interesting results presented by Brudzynski and coworkers [7,8,9,10], Bucekova et al., [11] and Grecka and colleagues [12] suggested an important role of plant-derived phytochemicals (mostly polyphenols) for the level of production of hydrogen peroxide in some honey types and probably also the transformation of H_2_O_2_ for most active radical products, e.g., OH˙ [7,8,9,10,11,12]. A unique mechanism of antimicrobial activity has been identified for New Zealand’s manuka honey and several Australian and Malaysian honeys. High efficiency in the inhibition of bacterial growth by these product is attributed to a non-peroxide component—methylglyoxal [13,14,15]. 

Propolis is a highly agglutinative, resinous substance of complex chemical composition that is collected by bees from flower and leaf buds. Propolis-containing extracts exhibit a broad spectrum of biological activities, among which antimicrobial potential has been the most intensively investigated [3,4,16]. The research carried out in our research group revealed the high antibacterial—particularly anti-staphylococcal [17] and antifungal [18]—activity of propolis collected in Polish apiaries, and flavonoids (flavonols, flavones, and flavanones) have been identified as components crucial for the antimicrobial activity of these products. Many trials have confirmed usefulness of propolis-containing products (e.g., extracts, ointments, wound materials, and dental materials such as toothpaste, glass-ionomer cement (GIC), and dental varnish) for treatment and prophylaxis against bacterial and fungal infections [4,19]. Interesting health-associated properties including antimicrobial potential have also been identified and described for lesser known and less popular bee products, namely royal jelly [20,21], bee wax [22], and bee venom [23].

The bee products that have recently gained particular popularity are bee pollen (BP) and bee bread (BB) [5]. Because of their high nutrition values, both of them are classified as functional foods [24] and both exhibit a wide range of therapeutic properties, such as antimicrobial, antioxidant, anti-radiation, anti-inflammatory, anti-tumor, hepatoprotective, and chemopreventive/chemoprotective benefits [5,24,25,26,27]. The term “bee bread” refers to the collected pollen that is processed by bees and fermented [5]. The exact mechanism of the biotransformation of BP to BB is still not fully elucidated. However, it known that enzymes from bees’ glands (e.g., amylases that are responsible for starch hydrolysis), as well as bacteria (mostly lactic acid bacteria—LAB) and some yeasts sourced from bees’ saliva and surfaces of pollen loads, play crucial roles in BP fermentation and BB production [5,28,29,30]. Some of the BB is stored in the wells of the honeycomb through the winter, and in the spring it is used as a main source of proteins for the new populations of bee larvae. It has been also found that ethanolic or methanolic extracts of components of both BB and BP exhibit antimicrobial potential. The outcomes of our recent study revealed a considerably higher antimicrobial potential of extracts produced from BB compared to BP extracts [31]. We also found the efficient inhibition of growth of *Staphylococcus aureus* in water suspensions of both products [31].

Important gaps in our knowledge remain regarding the microbial ecosystem of bee products, including both bacteria and fungi. Still very little is known about species composition and the role of these microorganisms in maturing bee products (e.g., the biotransformation of BP to BB) and in the protection of honey and bee bread against microbial spoilage, which is crucial for the health of bees (both mature and larvae) and humans who consume these products. There is mounting evidence implicating microbial ecosystem of the bee raw materials (nectar and pollen)–bee products (honey and bee bread)–honey bee axis involved in the production of a range of antimicrobial agents. These agents are used as weaponry in competitive interspecies interactions to effectively kill competing microorganisms in the fight for nutrients and space in each of these niches (nectar, pollen, honey, bee bread, and honey bee). Among the secondary metabolites produced by microorganisms that constitute the microflora of bee products are antimicrobial peptides, bacteriocins, surfactants, siderophores, proteolytic enzymes, and cell wall-degrading enzymes [32]. The main goal of this study was to investigate the ability of bacteria that constitute the microbiome of BP and BB for the growth inhibition of selected pathogenic microorganisms. Most of isolated strains of bacteria were identified as *Bacillus* spp., and some of them exhibited high antagonistic activity against important clinical human pathogens including staphylococci, *E. coli*, and *P. aeruginosa*. Our future studies will be focused on the identification of the molecular mechanism or metabolites that are responsible for these antagonistic interactions. We believe this could lead to the identification of producers of new antimicrobial agents. Moreover, most of isolates derived from both raw materials revealed high proteolytic, lipolytic, esterolytic, and cellulolytic activity. The outcomes of the study revealed that bacteria isolated from BP and BB can be considered a possible source of novel antimicrobial compounds and enzymes of particular industrial importance.

## 2. Results

As is shown in Table 1, the investigated samples of BP and BB presented different, though generally low, levels of microbial contamination—only aerobic and facultative aerobic were considered in this study. Four products (44%), two samples of each BB and BP, exhibited a level of contamination of above 10^3^ CFU (colony forming units) per gram of the raw material. The other five samples contained less bacteria, from 100 to 600 CFU per gram of the raw material. No evident differences in the level of microbial contamination between BP and BB were observed in this study.

**Figure 1:**
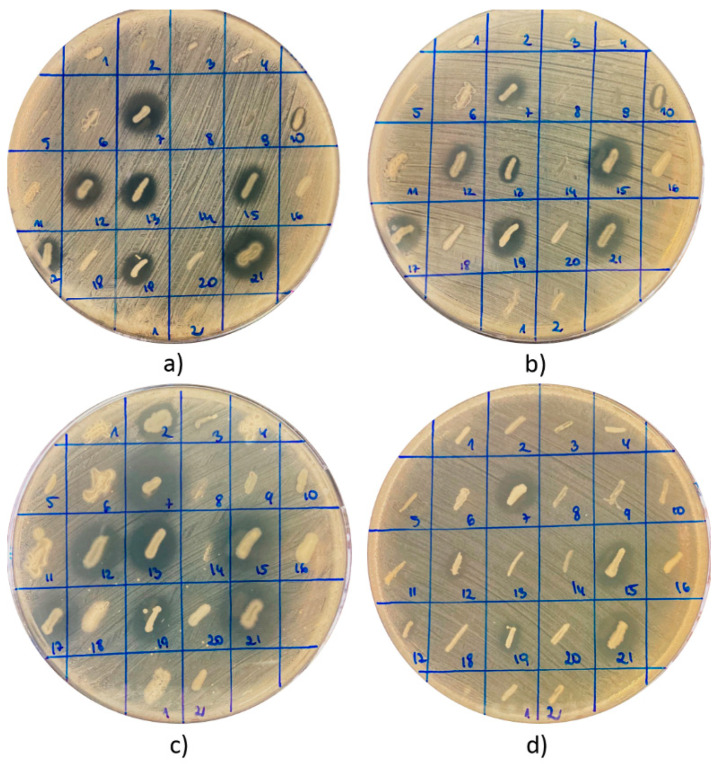
An example of antagonistic interaction between isolated strains (strains 1–21 were isolated from the product BB19, and strains 1 and 2 were derived from the product BB3) and indicatory/reference strains of pathogenic bacteria: (**a**) *S. aureus* ATCC 25923, (**b**) *S. aureus* ATCC 29213, (**c**) *S. epidermidis* ATCC 12228, and (**d**) *E. coli* ATCC 25922. Interactions with *P. aeruginosa* ATCC 27853 were a separately analyzed (results not presented).

In total, 81 strains of bacteria were recovered from nine tested products (Table 1 and Table 2). In each case, the bacteria were cultivated from a 0.1 mL suspension of the raw material in sterile water (1:10 *w/v*). All these isolates were screened for antagonistic interactions with pathogenic bacteria and the production of select essential hydrolytic enzymes. The antagonistic relationship was investigated through the observation of growth inhibition zones (GIZs) of indicator strains of bacteria around the growing colonies of tested strains—isolates from BB or BP (Figure 1).

Considering Gram-positive staphylococci, the antagonistic activity was observed for 27 (33.3%), 29 (35.8%), and 22 (27.2%) strains against *S. aureus* ATCC 25923, *S. aureus* ATCC 29213, and *S. epidermidis* ATCC 12228, respectively. A considerable number of strains, *n* = 32 (39.5%), inhibited the growth of *Pseudomonas aeruginosa* ATCC 27853, while activity against *E. coli* ATCC 25922 was not so common and was confirmed for 15 isolates (18.5%). The largest number of active isolates was recovered from bee bread assigned as BB19. Eight strains inhibited the growth of staphylococci, and an antagonistic relationship with *E. coli* ATCC 25922 and *P. aeruginosa* ATCC 27853 was confirmed for five (23.8%) and nine (42.9%) strains, respectively. Forty percent or more of isolates derived from products BP3, BP15, BP20, and BB10 exhibited antagonistic potential against both strains of *S. aureus* and *P. aeruginosa* ATCC 27853. On the other hand, the only strain recovered from the raw material was assigned as BP12, and both isolates from BB3 did not exhibit any antimicrobial properties. Relatively low percentage levels of active strains were also found in the cases of BB6 and BB15.

**Figure 2:**
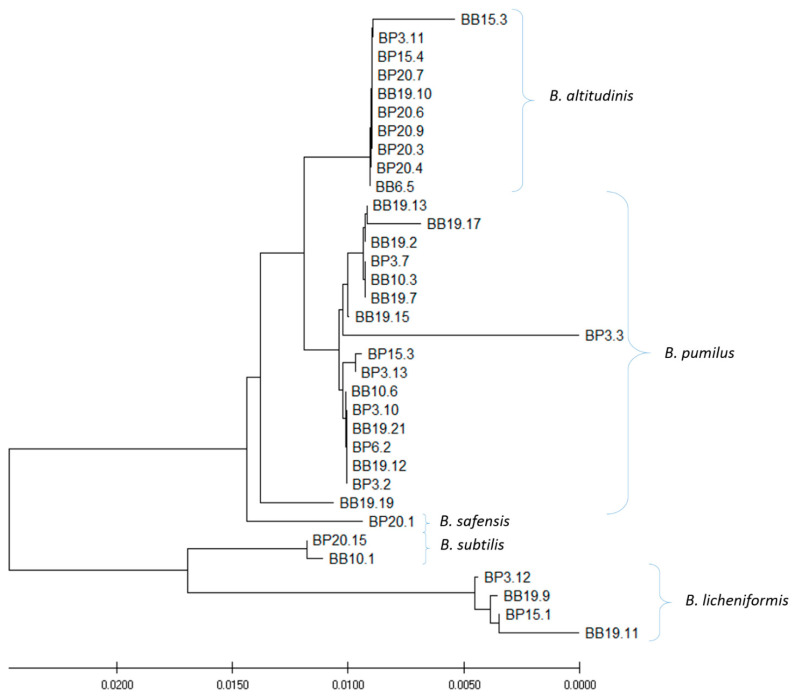
Phylogenetic tree of 34 strains that exhibited antagonistic potential against at least of one reference strain of pathogenic bacteria. MUSCLE multiple alignment/construction was done using the neighbor−joining method.

The 34 out of 81 isolated strains that exhibited antagonistic activity against at least one indicator strain were selected for species identification and amplification and sequencing of the gene coding for 16S rRNA (Table 2). The bioinformatics analysis of 16S rRNA gene sequences (carried out with the BLAST software) revealed that all tested strains belong to the genus *Bacillus*. In general, five species were distinguished among the isolates. Most of the strains (eighteen) were classified as *B. pumilus.* Nine isolates were identified as a second most common species—*B. altitudinis*. Four, two, and one strains were recognized as *B. licheniformis*, *subtilis,* and *safensis*, respectively. However, it is necessary to remember that sequences of the 16S rRNA gene of different species of *Bacillus* spp. are characterized by a high level of similarity or even identity. Thus, further analysis, e.g., whole genome sequencing or mass spectrometry, would be required for final species identification. The phylogenetic analysis based on results of comparative analysis of the sequences of 16S rRNA genes revealed some diversity between the tested strains (Figure 2) and generally confirmed results of the classification of the species (Figure 2).

Five clusters of the strains could be distinguished, which was in agreement with species identification.

**Figure 3:**
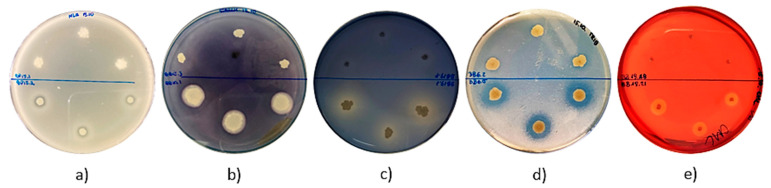
Enzymatic activity of isolates. Selected agar plates are presented with observed (**a**) proteolytic, (**b**) amylolytic, (**c**) lipolytic, (**d**) esterolytic, and (**e**) cellulolytic activity. In the upper part of the plates, there are strains that did not exhibit enzymatic activity, while in the lower part of the plates, there are strains capable of producing hydrolases.

Some interesting observations were also made regarding the assessment of the enzymatic activity of isolated strains of bacteria (Table 3 and Figure 3). None of the strains were able to produce laccase. No discoloration of the bacterial colonies was observed on the agar medium supplemented with guaiacol. Furthermore, a small number of strains (*n* = 3; 3.7%) exhibited amylolytic activity. Flooding the active strains with Lugol’s solution resulted in the appearance of clear halos around the colonies (Figure 3b). Eleven strains (13.58%) formed lightly blue colonies on the LA agar medium supplemented with X−gal, which probably confirmed β−galactosidase production, though with a low efficiency (data not shown). Almost half of the isolates presented esterolytic (*n* = 29; 36%) and proteolytic (*n* = 39; 48%) activity. In the case of strains isolated from BP and BB that are able to produce proteases and esterases, clear halos around the grown bacterial colonies were observed on the media supplemented with skimmed milk and tributyrin, respectively (Figure 3a,d). Moreover, a significant number of strains (*n* = 54; 67%) displayed cellulolytic activity, and 18 of them were classified as strong producers. In this case, the halos zones appeared around the colonies grown on LA agar medium supplemented with carboxymethylcellulose and flooded with Congo red solution (Figure 3e). Interestingly, the largest number of isolates (*n* = 69; 85.1%) exhibited lipolytic activity. The isolates contributed to the formation of bright halos around the colonies on Spirit Blue Agar (Figure 3c).

## 3. Discussion

The recently observed increase in the popularity and consumption level of BP and BB is mainly a consequence of the high nutritional value and health benefit properties of these products, including the high contents of vitamins, minerals, amino acids, some fatty acids, and polyphenols (antioxidants) that seem to be the most important [5,24,25,26]. However, very little is known about the microbiome of both these products. Both bacterial and fungal communities associated with BP and BB are important in at least four different aspects: (1) the process of biotransformation of BP to BB; (2) the stability of BB in the hive (in the cells of honey combs) during long term storage in the winter season; (3) the microbial safety of bees, particularly bee larvae that are fed with the BP/BB; and (4) the influence on the health of people who are consumers of BP/BB. The collected pollen loads by bee workers are prone to microbial deterioration—mostly due to molds. The BP that is sold in markets must be dried, which inhibits the growth of molds and other pathogenic microorganisms. For bees, the BP is, in fact, the only raw material for BB production. The BP loads collected by bee workers are mixed with small amounts of the secretion from the bee’s saliva, tightly packed in honeycomb cells, and finally covered with a thin layer of honey and a wax lid. Subsequently, under these anaerobic conditions, the BP undergoes the biotransformation process to BB. The exact biochemical mechanism of the biotransformation processes remains not fully understood. However, it is known that different enzymes from bees’ glandular secretion, as well as bacteria that are present in bees’ saliva and on the surface of pollen loads, are crucial for this process [5,33,34]. It still remains unclear which species of bacteria participate in BB maturing. However, the outcomes of several investigations have suggested that lactic acid bacteria are of primary importance. Vasquez and Olofsson (2009) observed the intense growth of these bacteria within maturing BB for about two weeks—the first step of BP biotransformation [30]. The LAB are important part of *Apis mellifera* gut community and are probably introduced to the raw material—BP from bees’ saliva [35]. The presence of LAB seems to be particularly important from the point of view of the microbial stability and preservation of the final product—BB. These bacteria produce lactic acid, bacteriocins, and aliphatic acids (products of lipids hydrolysis) that efficiently inhibit the growth of not only pathogenic (for both bees and humans, the consumers of BB) microorganisms but also bacteria, yeasts, and molds that could cause microbial deterioration or undesirable sensory changes [35]. Iorizzo and coworkers (2020) revealed the high inhibitory activity of LAB, namely *Lactobacillus kunkeei* and *Lactiplantibacillus plantarum* (isolated from bees’ gastrointestinal tract and bee products) against the important bee pathogens *Ascosphaera apis* and *Paenibacillus larvae*, respectively [36,37]. It also has been found that yeasts and molds participate in BP biotransformation. Detry et al. (2020) identified *Starmerella*, *Metschnikowia*, and *Zygosaccharomyces* as the most common yeast species in bee bread. However, the high abundance of yeasts in fresh bee bread decreased rapidly with the storage duration. *Starmerella* species dominated fresh bee bread, while mostly *Zygosaccharomyces* members were isolated from aged bee bread [38]. Disayathanoowat et al. (2020) investigated dynamic of bacterial and fungal community structures in corbicula pollen and hive−stored BB collected in China. They found that corbicula pollen was colonized by the *Enterobacteriaceae* bacterium (*Escherichia−Shigella*, *Panteoa*, and *Pseudomonas*) group; however, the number of bacteria significantly decreased in hive−stored bee bread in less than 72 h. In contrast, *Acinetobacter* was highly abundant and could utilize protein sources. In terms of the fungal community, the genus *Cladosporium* remained abundant in both corbicula pollen and hive−stored bee bread. The authors also concluded that filamentous fungus might encourage honey bees to reserve pollen by releasing organic acids [29].

Both mature BB and dried BP—ready for sale in markets—are considered microbial−safe and free from dangerous pathogenic microorganisms. However, none of these products are sterile, and very little is known about the microbiota of these products, including species composition and the metabolic and enzymatic properties of bacterial and fungal communities present in BP and BB. The herein presented results confirmed the generally low level of microbial contamination of samples of both products, with a maximum level of contamination of approximately 2.1 × 10^3^ CFU/g. Interestingly, all of 34 isolates that exhibited antimicrobial activity were classified into the genus *Bacillus* spp., and five different species were identified: *B. subtilis*, *B. licheniformis*, *B. pumilus*, *B*. *altitudinis,* and *B. safensis*. The above−mentioned studies did not show the presence of *Bacillus* spp. in BB samples [29,30,38]. In our opinion, this difference could be explained by the fact that only mature BB samples harvested from honeycomb cells and stored for about four months (under refrigeration) and dried BP samples were used in our study. The phytochemicals present in the raw material and in honey added to the BP, as well as the metabolites of bacteria growing in the maturing product, formed an unfavorable environment for the growth and development of most microorganisms. Thus, only highly resistant bacteria, e.g., spore−forming *Bacillus* spp. bacteria, can survive under these conditions. The outcomes of our previous investigation revealed very similar species composition and properties of microorganisms isolated from honey samples [39]. Most of these isolates were classified as *Bacillus* spp., and most of them exhibited the ability to produce metabolites of antibacterial activity [39]. One of these strains, namely *Paenibacillus alvei* MP1, was found to be an efficient producer of proteinaceous agent that exhibited promising activity against a broad spectrum of pathogenic bacteria [40]. Moreover, several genes responsible for antimicrobial activity have been identified in the genome of *P. alvei* MP1 [41]. Some other research groups have also reported the isolation of antimicrobials producing bacteria from honey. Lee et al. (2008) screened six US honeys and two manuka honeys originating from New Zealand. The researchers reported that 92.5% of a total of 2398 strains exhibited antimicrobial activity [42]. One of the isolates, identified as *Paenibacillus polymyxa,* showed a broad range of antibacterial activity against Gram−positive and −negative bacteria including *P. larvae* ssp. larvae ATCC 25747 and foodborne pathogens such as *Bacillus cereus* F4552 and *Escherichia coli* O157:H7 ATCC 43895 [43]. Zulkhairi Amin and coworkers (2020) revealed probiotic properties, including the production of antibacterial metabolites, of *Bacillus* spp. strains isolated from honey of the stingless bee *Heterotrigona itama* [44]. Khalili Samani et al. (2021) isolated several bacteriocin−producing strains of *Bacillus* spp. and Gram−positive cocci from the samples of Iranian honey. In contrast to the bacteriocins produced by these isolates, most of produced metabolites characterized in this study that were BP− and BB−derived strains that exhibited activity against both Gram− positive and Gram−negative bacteria [45]. However, to date, *Bacillus* spp.producing antimicrobial agents have not been isolated from BB or BP. A small amount of honey is added to BP before biotransformation, and this could be the source of *Bacillus* spp. in the final product—BB. On the other hand, bacteria of the genus *Bacillus* are common in the environment and could be present on the surfaces of pollen grains collected by bee workers.

Important and interesting information provided in this study included the investigation of enzymatic potential of the isolates. The production of lipases, cellulases, and proteinases were most common among tested strains. It can be assumed that these activities were essential for the “extraction” of basic food ingredients: amino acids, fatty acids, and glucose from components of pollen grains—proteins, lipids, and cellulose. These activities also improve the nutritional value of BB through the pre−digestion of biopolymers (e.g., cellulose and proteins), which is important for bees and human consumers. Moreover, the release of aliphatic acids from lipids can be important for the preservation of BB. Markiewicz−Żukowska and coworkers (2013) identified aliphatic acids as important antimicrobial components of BB, and unsaturated, α−linolenic, linoleic, oleic, and 11,14,17−eicosatrienoic acids formed more than a half of them (40.63 ± 4.5%) [46]. Neither β−galactosidase nor laccase are crucial for surviving in BB or BP, so these activities are rarely observed or not observed at all. Surprisingly, a relatively low percentage level of alpha amylase positive isolates was identified. Starch is important component of BP, and only 3 out of 81 tested strains exhibited strong potential for the hydrolysis of this polysaccharide. To our knowledge, the enzymatic potential of bacteria, other than LAB, isolated from BB or BP had not been investigated to date. Most of bacteria that belong to the genus *Bacillus* are not harmful to mammalians, with the exception of *B. cereus* and *B. anthracis*. Thus, the strains isolated from bee products, including BB or BP, can be considered sources of antimicrobials or enzymes. Moreover, they are also suitable candidates for probiotic bacteria [44].

## 4. Materials and Methods

### 4.1. Essential Chemical Reagents and Growth Media

All chemicals and growth media were purchased from commercial sources. LB broth and LB agar medium were bought from A&A Biotechnology (Gdynia; Poland). Mannitol salt phenol−red agar, Spirit blue agar, tributyrin agar, skimmed milk, starch, carboxymethylcellulose, guaiacol, X−gal (5−bromo−4−chloro−3−indolyl−β−D−galactopyranoside), Tween 80, cottonseed oil, Lugol’s solution, Congo red dye, and PBS tablets (pH 7.4) were purchased from Merck (Darmstadt, Germany). Ultrapure H_2_O (18.0 MΩ) was produced with the Milli−Q Advantage A10 system (Millipore, Billerica, MA, USA).

### 4.2. Bee Pollen and Bee Bread Samples and Isolation of Bacterial Strains

The samples of bee pollen (*n* = 4) and bee bread (*n* = 5) were provided by Polish apiaries. All samples of BP were dried (to protect the product against microbial spoilage). The BB samples were directly recovered from honeycombs in late summer or autumn 2019; thus, only mature bee bread was used for the study. All products were not older than six months counting from the date of harvesting to the date of using them for the experiment. The samples of BP were stored in dark conditions at ambient temperature, and BB was kept refrigerated at 4 °C. The suspensions of BP and BB in sterile deionized water at a 1:10 (*w/v*) ratio were performed for sample preparation. Subsequently, 100 µL of each suspension were streaked on the LB (Luria–Bertani) agar medium. The plates with inoculated agar medium were incubated at 37 °C for 24 h. Thereafter, the growing colonies were enumerated, and the level of microbial contamination of BB and BP samples (CFU/g of the product) was calculated. Each colony was individually transferred onto new Petri dish with an LB agar medium and incubated overnight at 37 °C. Then, a collection of isolates from BP and BB was obtained for further investigation.

### 4.3. Growth Inhibitory Assay

For assessing the antimicrobial activity of isolated bacteria, the colonies from the collection were transferred with sterile pipette tips onto LB agar plates inoculated with reference strains: *S. aureus* ATCC 253923, *S. aureus* ATCC 29213, *S. epidermidis* ATCC 12228, *E. coli* ATCC 25922, and *P. aeruginosa* ATCC 27853. Reference indicator strains were inoculated using a sterile cotton swab soaked in a diluted suspension of each tested strain prepared in a phosphate buffered solution (final optical density of each solution OD_600_ = 0.1; approximately 1–5 × 10^8^ CFU/mL). Agar plates were incubated for 24 h at 37 °C. Thereafter, the presence and size of the GIZs of indicator strains were observed and recorded. The antimicrobial activity of each isolate was determined on the basis of the sizes of growth inhibition zones of indicatory strains observed around the colonies of bacteria isolated from BP or BB. All tests were performed in at least triplicate. Isolated bacteria with antimicrobial activity against reference strains were cataloged for further investigation.

### 4.4. Investigation of Enzymatic Activity of Isolated Strains

The bacterial isolates from BP and BB were tested to confirm or exclude their ability to produce enzymes from the group of hydrolases such as proteases, cellulases, amylases, esterases, lipases, laccases, and β−galactosidases. Two aspects were taken into account for the selection of the set of enzymatic activities that were investigated: the chemical composition of the BP (and therefore the substances that are available for the bacteria) and the industrial relevance of the enzymes regarding their application in industry. In order to determine the individual hydrolytic activities of isolates, the following media were applied: LB agar with skimmed milk (1.5% *w/v*) for proteolytic activity, LB agar with carboxymethylcellulose (2% *w/v*) for cellulolytic activity, LB agar with starch (2% *w/v*) for amylases activity, LB agar with guaiacol (100 µL/L) for laccase activity, LB agar with X−gal (20 mg/L) for β−galactosidase activity, a Tributyrin agar with neutral tributyrin (10 g/L) for esterolytic activity and a Spirit blue agar supplemented with 30 mL/L of lipase substrate (400 mL of warm distilled water, 1 mL of Tween 80, and 100 mL of cottonseed oil) for lipase activity. Using sterile pipette tips, each of isolates was applied on the appropriate agar medium in triplicate and incubated for 24 h at 37 °C. Following incubation, the appearance of halos around the colonies was observed for the confirmation of proteinase, esterase, and lipase activities. Halo zones around colonies indicated amylases and cellulolytic activities. However, the confirmation of the production of these enzymes required the flooding of the agar medium with Lugol’s solution or Congo red solution, respectively. Staining the growing colonies with blue or brownish dye was able to confirm the production of beta−galactosidase or laccase, respectively.

### 4.5. Identification of Bacterial Species of Isolates That Exhibited Antagonistic Activity against Selected Pathogenic Microorganisms

The identification of the isolates that exhibited antagonistic activity against selected pathogenic bacteria was executed by sequencing of the 16S rRNA gene. The DNA was isolated using Genomic Mini AX Bacteria+ (A&A Biotechnology, Gdynia, Poland) according to the protocol purchased from the manufacturer of the kit.

The PCR amplification of the targeted gene was determined with a pair of primers:

rP1 5′ CCCGGGATCCAAGCTTAGAGTTTGATCCTGGCTCAG 3′

Fd2 5′ CCCAATTCGTCGACAACACGGCTACCTTGTTACGACTT 3′

The amplified products sequencing was carried out by Macrogen (Amsterdam, the Netherlands). The purification of the amplified gene coding for 16S rRNA was executed using the enzymatic Post−PCR Immediate Cleanup (EPPiC) purification kit (A&A Biotechnology, Gdynia, Poland) following the protocol provided by the producer.

### 4.6. DNA Sequence Analysis

BLAST (Basic Local Alignment Search Tool) was used to the sequence analyses. Multiple sequence alignment in the MEGA X software was performed using the MUSCLE algorithm. The phylogenetic tree was constructed from the 16S rRNA sequences from previously generated FASTA sequence using the MEGA X software. The phylogenetic tree was assembled using the neighbor−joining method and sorting by distance.

## 5. Conclusions

Bacteria of the genus *Bacillus* have been identified as most important component of mature and stored BB, as well as dried BP. Moreover, the outcomes of the study revealed that BP and BB can be considered to be potential sources of bacteria producing antimicrobial agents and/or enzymes of particular industrial importance. Of course, additional research is required to verify this hypothesis, but the results of preliminary studies are promising.

## Figures and Tables

**Table 1:** Level of microbial contamination of investigated BP and BB samples and the antagonistic activity of isolates against reference strains of pathogenic bacteria.

Sample	No. of Colonies	CFU/g of Product	Activity against *S. aureus* ATCC 25923		Activity against *S. aureus* ATCC 29213		Activity against *S. epidermidis* ATCC 12228		Activity against *E. coli* ATCC 25922		Activity against *P. aeruginosa* ATCC 27853	
			No. of Colonies	%	No. of Colonies	%	No. of Colonies	%	No. of Colonies	%	No. of Colonies	%
BP3	14	1400	6	42.86	6	42.86	5	35.71	5	35.71	7	50.00
BP15	5	500	2	40.00	2	40.00	2	40.00	2	40.00	3	60.00
BP12	1	100	0	0.00	0	0.00	0	0.00	0	0.00	0	0.00
BP20	15	1500	6	40.00	7	46.67	3	20.00	1	6.67	7	46.67
BB3	2	200	0	0.00	0	0.00	0	0.00	0	0.00	0	0.00
BB6	13	1300	1	7.69	2	15.38	1	7.69	1	7.69	2	15.38
BB10	6	600	3	50.00	3	50.00	3	50.00	1	16.67	3	50.00
BB15	4	400	1	25.00	1	25.00	0	0.00	0	0.00	1	25.00
BB19	21	2100	8	38.10	8	38.10	8	38.10	5	23.81	9	42.86
TOTAL	81		27	33.33	29	35.80	22	27.20	15	18.52	32	39.51

**Table 2:** Species classification based on the BLAST analysis of 16S rRNA gene sequences and antagonistic activity against reference strains of pathogenic bacteria.

Sample	Species Classification *	Exhibited Activity				
		*S. aureus* ATCC 25923	*S. aureus* ATCC 29213	*S. epidermidis* ATCC 12228	*E. coli* ATCC 25922	*P. aeruginosa* ATCC 27853
BP3.2	*Bacillus* spp. (*pumilus, zhangzhouensis*)	++	+++	+++	+++	+++
BP3.3	*Bacillus* spp. *(pumilus, zhangzhouensis*)	+	++	++	++	++
BP3.7	*Bacillus* spp. (*pumilus, zhangzhouensis*)	+	+	+	++	++
BP3.10	*Bacillus* spp. (*pumilus, zhangzhouensis*)	+++	+++	+++	+++	+++
BP3.11	*Bacillus* spp. (*altitudinis, stratosphericus*)	+	+	−	−	+++
BP3.12	*Bacillus* spp. (*licheniformis, aerius*)	−	−	−	−	++
BP3.13	*Bacillus* spp. (*pumilus, zhangzhouensis*)	+++	+++	+++	+++	+++
BP15.1	*Bacillus* spp. (*lichemiformis*)	−	−	−	−	+++
BP15.3	*Bacillus* spp. (*pumilus, zhangzhouensis*)	++	++	+++	+++	+++
BP15.4	*Bacillus* spp. (*altitudinis, stratosphericus*)	+++	+++	+++	+++	++
BP20.1	*Bacillus* spp. (*safensis, pumilus*)	+	+	−	−	++
BP20.3	*Bacillus* spp. (*altitudinis, stratosphericus*)	+	+	−	−	++
BP20.4	*Bacillus* spp. (*altitudinis, stratosphericus*)	+	+	−	−	+
BP20.6	*Bacillus* spp. (*altitudinis, stratosphericus*)	−	+	−	−	+++
BP20.7	*Bacillus* spp. (*altitudinis, stratosphericus*)	+	+	+++	−	++
BP20.9	*Bacillus* spp. (*altitudinis, stratosphericus*)	+++	++	++	++	++
BP20.15	*Bacillus* spp. (*subtilis*)	+++	++	+	−	+++
BB6.2	*Bacillus* spp. (*pumilus, zhangzhouensis*)	+++	++	+++	++	++
BB6.5	*Bacillus* spp. (*altitudinis, stratosphericus*)	−	++	−	−	+
BB10.1	*Bacillus* spp. (*subtilis*)	+++	+++	+++	−	+++
BB10.3	*Bacillus* spp. (*pumilus, zhangzhouensis*)	+	+	+	−	++
BB10.6	*Bacillus* spp. (*pumilus, zhangzhouensis*)	+++	+++	+++	+++	+++
BB15.3	*Bacillus* spp. (*altitudinis, stratosphericus*)	+	++	−	−	+++
BB19.2	*Bacillus* spp. (*pumilus, zhangzhouensis*)	−	−	++	−	−
BB19.7	*Bacillus* spp. (*pumilus, zhangzhouensis*)	+++	++	+++	+++	+++
BB19.9	*Bacillus* spp. (*licheniformis, aerius*)	−	−	−	−	++
BB19.10	Bacillus spp. (*altitudinis, aerius*)	+	+	−	−	+
BB19.11	*Bacillus* spp. (*licheniformis, paralicheniformis*)	−	−	−	−	+++
BB19.12	*Bacillus* spp. (*pumilus, zhangzhouensis*)	++	++	+++	+	+++
BB19.13	*Bacillus* spp. (*pumilus, zhangzhouensis*)	+++	++	+++	−	++
BB19.15	*Bacillus* spp. (*pumilus, zhangzhouensis*)	++	+++	+++	+++	+++
BB19.17	*Bacillus* spp. (*pumilus, zhangzhouensis*)	+++	+++	++	−	+++
BB19.19	*Bacillus* spp. (*pumilus, zhangzhouensis*)	+++	+++	++	++	−
BB19.21	*Bacillus* spp. (*pumilus, zhangzhouensis*)	+++	+++	+++	+++	+++

*—sequences of gene coding for 16S rRNA of different species of the genus *Bacillus* exhibit high level of similarity. Thus, in most cases, two most possible species are proposed. The classification of antagonistic interaction as S—strong (+++); M—moderate (++); W—weak (+); or L—lack (−) was based on the measurement of the size of the growth inhibition zone (SGIZ) of indicatory strain counted from the edge of the colony of the investigated isolate. The following scale was used for the classification of antagonistic interactions: strong—SGIZ > 3 mm; moderate—SGIZ in the range from 1 to 3 mm; and weak—SGIZ ≤ 1 mm. The mean value of this parameter from three independent experiments was used for final classification of each strain tested.

**Table 3:** Enzymatic activity of isolates.

	Isolates	Proteolytic Activity	Amylolytic Activity	Lipolytic Activity	Esterolytic Activity	Cellulolytic Activity	Presence of Beta−Galactosidase	Presence of Laccase
1	BP3.1	−	−	−	−	−	−	−
2	BP3.2	+++	−	+	−	++	−	−
3	BP3.3	++	−	++	−	++	−	−
4	BP3.4	−	−	−	−	−	−	−
5	BP3.5	+++	−	++	++	+++	−	−
6	BP3.6	+	−	+	−	+	−	−
7	BP3.7	+	−	++	++	−	−	−
8	BP3.8	−	−	−	−	−	−	−
9	BP3.9	−	+++	++	++	+++	+	−
10	BP3.10	+++	−	++	−	++	−	−
11	BP3.11	+	−	+	−	+	−	−
12	BP3.12	−	−	+++	+	+++	+	−
13	BP3.13	++	−	+	−	++	−	−
14	BP3.14	+	−	+	−	+	−	−
15	BP12.1	++	−	+++	−	++	+	−
16	BP15.1	−	−	+++	−	++	−	−
17	BP15.2	++	−	+	+	++	−	−
18	BP15.3	+++	−	++	−	++	−	−
19	BP15.4	+	−	++	−	+++	−	−
20	BP15.5	−	−	++	−	−	−	−
21	BP20.1	++	−	++	−	−	−	−
22	BP20.2	++	−	+++	−	+	+	−
23	BP20.3	++	−	++	+	+++	−	−
24	BP20.4	++	−	++	+	++	−	−
25	BP20.5	−	−	+++	−	+	−	−
26	BP20.6	+++	−	++	+	++	−	−
27	BP20.7	++	−	++	+	++	−	−
28	BP20.8	++	−	+++	−	−	+	−
29	BP20.9	++	−	++	+	+++	−	−
30	BP20.10	++	−	−	−	−	−	−
31	BP20.11	−	−	+	−	+	+	−
32	BP20.12	−	−	+	++	−	−	−
33	BP20.13	−	−	−	−	−	−	−
34	BP20.14	−	−	−	−	−	−	−
35	BP20.15	+++	+++	+++	+	+++	−	−
36	BB3.1	−	−	++	−	+++	−	−
37	BB3.2	−	−	−	−	−	−	−
38	BB6.1	−	−	++	−	−	+	−
39	BB6.2	+++	−	++	+	++	−	−
40	BB6.3	−	−	++	−	−	+	−
41	BB6.4	−	−	−	−	−	−	−
42	BB6.5	−	−	+++	+++	++	−	−
43	BB6.6	−	−	−	−	−	−	−
44	BB6.7	−	−	+	−	−	−	−
45	BB6.8	++	−	++	−	+	+	−
46	BB6.9	−	−	+++	−	−	−	−
47	BB6.10	−	−	−	−	−	−	−
48	BB6.11	−	−	+	++	+++	−	−
49	BB6.12	−	−	+++	−	+++	−	−
50	BB6.13	−	−	++	+	++	−	−
51	BB10.1	+	+++	+	++	+++	−	−
52	BB10.2	−	−	+++	−	++	−	−
53	BB10.3	+	−	++	++	+	−	−
54	BB10.4	−	−	+++	−	++	−	−
55	BB10.5	−	−	+++	−	+++	+	−
56	BB10.6	++	−	+	++	++	−	−
57	BB15.1	−	−	+	+	+	−	−
58	BB15.2	−	−	+	−	+	−	−
59	BB15.3	+	−	+	+	++	−	−
60	BB15.4	−	−	+++	−	+++	−	−
61	BB19.1	−	−	+++	−	++	−	−
62	BB19.2	+++	−	++	+	+	−	−
63	BB19.3	−	−	++	−	−	−	−
64	BB19.4	−	−	++	−	−	−	−
65	BB19.5	−	−	++	+	−	−	−
66	BB19.6	+	−	++	−	++	−	−
67	BB19.7	+	−	+++	+	++	−	−
68	BB19.8	−	−	++	−	−	−	−
69	BB19.9	−	−	−	+	+	−	−
70	BB19.10	++	−	++	−	+++	−	−
71	BB19.11	−	−	+	−	+	+	−
72	BB19.12	+++	−	++	+	+++	−	−
73	BB19.13	++	−	++	+	++	−	−
74	BB19.14	−	−	+	−	−	−	−
75	BB19.15	+	−	++	−	+++	−	−
76	BB19.16	−	−	−	−	−	−	−
77	BB19.17	++	−	+++	−	+++	−	−
78	BB19.18	−	−	++	−	−	−	−
79	BB19.19	++	−	+++	+	−	−	−
80	BB19.20	−	−	++	−	++	−	−
81	BB19.21	+++	−	+++	+	+++	−	−
TOTAL								
S		10 (12%)	3 (4%)	19 (23%)	1 (1%)	18 (22%)	0 (0%)	0
M		18 (22%)	0	33 (41%)	8 (10%)	23 (28%)	0 (0%)	0
W		11 (14%)	0	17 (21%)	20 (25%)	13 (16%)	11 (14%)	0
L		42 (52%)	78 (96%)	12 (15%)	52 (64%)	27 (33%)	70 (86%)	81 (100%)

S—strong (+++); M—moderate (++); W—weak (+); L—lack (−).

## Data Availability

The data presented in this study are available on request from the corresponding author.
